# Multidimensional Evaluation of Virtual Reality Paradigms in Clinical Neuropsychology: Application of the VR-Check Framework

**DOI:** 10.2196/16724

**Published:** 2020-04-27

**Authors:** Stephan Krohn, Johanne Tromp, Eva M Quinque, Julia Belger, Felix Klotzsche, Sophia Rekers, Paul Chojecki, Jeroen de Mooij, Mert Akbal, Cade McCall, Arno Villringer, Michael Gaebler, Carsten Finke, Angelika Thöne-Otto

**Affiliations:** 1 Department of Neurology Charité-Universitätsmedizin Berlin Berlin Germany; 2 Berlin School of Mind and Brain Humboldt-Universität zu Berlin Berlin Germany; 3 Max-Planck Institute for Human Cognitive and Brain Sciences Leipzig Germany; 4 Clinic for Cognitive Neurology University Hospital Leipzig Leipzig Germany; 5 Fraunhofer Institute for Telecommunications Heinrich Hertz Institute Berlin Germany; 6 Hochschule der Bildenden Künste Saar Saarbrücken Germany; 7 Department of Psychology University of York York United Kingdom

**Keywords:** virtual reality, neuropsychology, cognition, research design

## Abstract

Virtual reality (VR) represents a key technology of the 21st century, attracting substantial interest from a wide range of scientific disciplines. With regard to clinical neuropsychology, a multitude of new VR applications are being developed to overcome the limitations of classical paradigms. Consequently, researchers increasingly face the challenge of systematically evaluating the characteristics and quality of VR applications to design the optimal paradigm for their specific research question and study population. However, the multifaceted character of contemporary VR is not adequately captured by the traditional quality criteria (ie, objectivity, reliability, validity), highlighting the need for an extended paradigm evaluation framework. To address this gap, we propose a multidimensional evaluation framework for VR applications in clinical neuropsychology, summarized as an easy-to-use checklist (VR-Check). This framework rests on 10 main evaluation dimensions encompassing cognitive domain specificity, ecological relevance, technical feasibility, user feasibility, user motivation, task adaptability, performance quantification, immersive capacities, training feasibility, and predictable pitfalls. We show how VR-Check enables systematic and comparative paradigm optimization by illustrating its application in an exemplary research project on the assessment of spatial cognition and executive functions with immersive VR. This application furthermore demonstrates how the framework allows researchers to identify across-domain trade-offs, makes deliberate design decisions explicit, and optimizes the allocation of study resources. Complementing recent approaches to standardize clinical VR studies, the VR-Check framework enables systematic and project-specific paradigm optimization for behavioral and cognitive research in neuropsychology.

## Introduction

Over the past few decades, virtual reality (VR) has emerged as one of the most rapidly advancing technologies of the 21st century, attracting substantial attention from a variety of scientific disciplines, including neuroscience. VR may be regarded as an umbrella term subsuming the real-time presentation of a computer-generated environment to a human user. Users perceive the environment through visual or multisensory stimulation and interact with it through reciprocal data exchange with the computer system, such that VR represents an advanced form of human-computer interaction [1]. VR can be broadly categorized into nonimmersive applications⸺2-dimensional (2D) screen presentations with interaction devices such as a keyboard or a joystick⸺and immersive applications that are more complex and require the integration of computers with further devices such as head-mounted displays (HMDs), VR controllers, or body-tracking sensors. These immersive systems enable users to experience the virtual environment concealed from the outside world and interact with it based on head or body movements.

**Figure 1:**
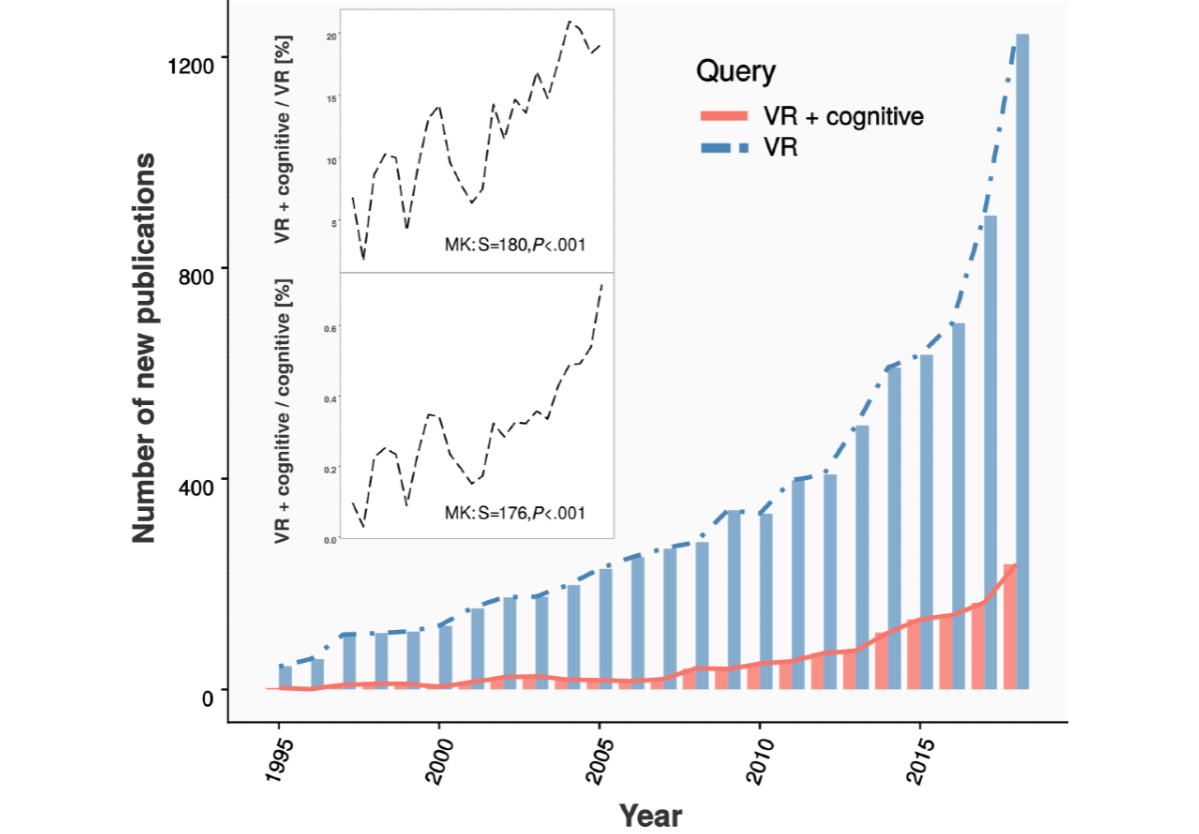
Temporal trends in the biomedical virtual reality literature. The PubMed database was searched for unique novel publications in the years 1995-2018 with the queries “Virtual Reality” (VR), “Virtual Reality” AND “cognitive” (VR + cognitive), and “cognitive.” Absolute new publication numbers for the former 2 queries are displayed as bars (search: September 2019). As absolute publications rose for both the VR and cognitive query, we computed the respective ratios of publication numbers over time, as shown in the inset. The proportion of annual VR + cognitive PubMed hits over all VR PubMed hits has risen to approximately 20% over the last 20 years, and nonparametric Mann-Kendall (MK) trend analysis indicates a monotonic upward trend of this proportion (S: sample estimate; positive numbers indicate upward trend). A similar temporal trend was observed for the ratio of VR + cognitive over all cognitive PubMed hits, although this proportion remains well under 1%.

In the context of developing paradigms for clinical research, VR provides scientists with a unique combination of extensive design possibilities and strong experimental control. Consequently, VR-based approaches are increasingly being pursued in biomedical research and specifically with respect to investigating cognitive function with VR (Figure 1). As a result, a fast-growing number of neuropsychological VR paradigms are being developed [1-12], paralleled by decreasing costs of hardware components and the increasing availability of open-access software systems for creating new VR paradigms in a customized manner [13-16]. Although these advancements open up many opportunities to investigate the clinical potential of VR, they increasingly present researchers with the challenge of defining the optimal paradigm to answer the research question at hand and leverage the advantages of the technology. Screening the VR literature for suitable paradigms, for instance, how should one evaluate the strengths and weaknesses of a particular paradigm, weigh them against each other, and systematically compare quality across several candidate tasks? Similarly, when developing an experimental VR paradigm de novo, what task features are important to consider in the design process, which qualities should an *ideal* VR task possess, and are there trade-offs in these qualities on which a deliberate design decision must be made?

In this methodological viewpoint paper, we propose a pragmatic framework to address these questions and advance the development of VR-based research tools. To motivate our approach, we first review task evaluation based on the traditional psychometric quality criteria. We contrast these endeavors with the extensive degrees of freedom in state-of-the-art VR, illustrating that the traditional quality criteria alone are inadequate to capture the multifaceted nature of VR paradigms comprehensively. To overcome this gap, we propose a general and multidimensional evaluation framework for neuropsychological VR paradigms in the form of a checklist (VR-Check), and we illustrate the application of this framework in a concrete research project. In the following sections, we focus on VR paradigms for neuropsychological assessment, rather than rehabilitation or cognitive training paradigms. Whereas many of the VR-Check dimensions will be equally relevant to training and rehabilitation tools, we here avoid a conflation of diagnostic and therapeutic VR applications for clarity.

## Evaluation Criteria in Classical Neuropsychological Tasks

Neuropsychological assessment tools have a long-standing history in clinical neuropsychology, with several tasks still widely in use more than half a century after their initial presentation (eg, the Wisconsin Card Sorting Test [17,18], or the Stroop Test [19,20]). Early work before the advent of neuroimaging was primarily driven by the aim to measure closely defined cognitive constructs with a clear link to specific brain areas to answer diagnostic questions not otherwise solvable at the time [21]. These early tests were predominantly evaluated according to the traditional psychometric quality criteria [22]. In brief, test results had to be independent of the experimenter (objectivity), consistently reproducible over repeated measurements (reliability), and should measure the intended construct (validity quality demands that are still widely accepted in cognitive psychology today.

With the introduction of neuroimaging into routine diagnostics, however, the mandate for clinical neuropsychologists has changed. Rather than helping to identify the neuroetiology, neuropsychologists are now faced with requests to predict and rehabilitate everyday functions, calling for a new type of paradigm tailored to do so [21]. In consequence, the need for an additional evaluation criterion, which better captures the relationship of the neuropsychological paradigm to everyday functioning, has been discussed for some time [1,21,23-26]. This relationship has been subsumed under the label *ecological relevance* [1,23]. Although in itself still subject to conceptual refinements, ecological relevance is commonly understood to posit that tests should capture the cognitive demands of daily life as closely as possible, resulting in high face validity [27], increased sensitivity to neurorehabilitation, and improved predictive power for everyday functioning [26]. One landmark publication of a test following these principles is a 1991 paper introducing the Multiple Errands Test (MET) [28] to measure multitasking. The MET comprises a list of shopping-related errands to be performed as a real-life task (ie, in a real shopping mall). Although the test features high ecological relevance, its limitations include reduced objectivity and reliability owing to unforeseeable variations in the real-world mall, high demand for resources to accompany patients in the environment, and not least safety issues and inapplicability to patients with more severe disabilities [29]. Although theoretically appealing, real-life tasks have therefore not entered routine neuropsychological assessment and are unlikely to do so due to the lack of control over the test environment. In sum, the search for ecologically relevant, yet experimentally well-controlled tasks is still very much ongoing. In this aspect, VR has the potential to facilitate crucial progress in the field.

## Overcoming the Limitations of Classical Tasks With Virtual Reality

Creating a virtual world offers many degrees of freedom: from the environment itself to the objects in that environment, and even the physics that govern the world. It is therefore possible to design environments that resemble the real world and its demands much more closely than routine paper-and-pencil tests. At the same time, VR preserves strong control over the experimental conditions (eg, the existence, type, and frequency of distractors, which are uncontrollable in real-life tasks such as the MET). Similarly, safety concerns of real-world tasks are attenuated by VR paradigms, as patients are not exposed to actual physical dangers (eg, Navarro et al [30] who used VR to test the act of crossing the street in stroke patients with neglect). Another advantage concerns the increased flexibility of the paradigm development itself: task modifications are implemented computationally, enabling a task design that specifically caters to the study population under question, the research question of interest, or an individual patient’s needs. This increased flexibility also illustrates a further limitation of many classical neuropsychological tasks: the lack of parallel versions. In virtual environments, in contrast, parallel task versions are much more easily created by computational modification. Furthermore, routine neuropsychological assessment is highly personnel dependent, requiring substantial resources in terms of patient assistance and monitoring. In addition, the evaluation of behavioral performance in classical assessment tasks usually requires time-consuming processing and examination of numeric data (eg, calculating scores), which then have to be visualized in a graph or table [1]. In real-life tasks such as the MET, acquisition and evaluation of performance data are even more challenging, as a trained professional has to attend to the patient continuously. VR-based assessment, in contrast, allows for the automatic generation of standardized test scores and reduces the demand for monitoring resources during an assessment. Performance evaluation can be augmented by intuitive feedback to the user (eg, playback), which may be especially beneficial for certain age groups or patient populations [5,8,31]. Finally, the personnel dependence of traditional approaches constitutes one factor limiting the widespread availability of high-quality neuropsychological care (eg, in more rural areas or in patients with restricted mobility). In contrast, VR systems can be employed in patients’ homes, offering a long-term perspective of improved ambulatory care and telerehabilitation.

## Evaluation Criteria in Neuropsychological Virtual Reality Paradigms

These advantages of VR raise hopes to ameliorate some of the limitations inherent to classical neuropsychological paradigms. However, they also illustrate the multitude of features over which VR paradigms can vary. Currently, neuropsychological VR paradigms are still to be evaluated in the light of the traditional quality criteria, although the latter were initially developed for a fundamentally different kind of assessment, commonly based on paper-and-pencil tests. In general, the traditional psychometric quality criteria remain valid for newly developed tests, including VR paradigms. Nonetheless, along with the increased design possibilities of contemporary VR, new evaluation dimensions emerge above and beyond these classical criteria, highlighting the need for an extended evaluation framework to capture the multidimensional nature of VR applications more adequately. Below, we propose such an evaluation framework that allows for systematic and comparative optimization of VR paradigms in clinical neuropsychology.

## VR-Check: Multidimensional Evaluation of Virtual Reality Paradigms

The framework rests on 10 evaluation dimensions, each comprising several subfeatures. These evaluation criteria are summarized in the form of a checklist (VR-Check; see below).

### Domain Specificity

This evaluation dimension examines how closely the cognitive domain of interest is targeted by the candidate paradigm. This aspect is especially relevant to VR paradigms, as they differ markedly from classical tasks in both clinical and experimental paradigms: The former usually involves a paper-and-pencil test with task instruction, execution, and evaluation by a trained professional. The latter typically involves the well-controlled presentation of predefined stimuli on a 2D computer screen and the measurement of a predefined set of responses, commonly assessed by interaction devices such as a mouse or a keyboard. In both settings, stimulation is rather unisensory, and participants are limited in their ability to act outside the predefined test space. In contrast, VR allows for increased degrees of behavioral freedom, commonly including the liberty to explore the test environment. Compared with classical tasks in neuropsychology, VR furthermore permits a much higher level of self-initiated action and interactivity as well as the possibility of multisensory stimulation. Although this underscores one particular strength of the technology, these increased degrees of freedom may also recruit other cognitive domains than the one we would like to target. This can make it difficult to ascribe differences in task performance to differences in the cognitive domain under study. Therefore, a VR candidate paradigm should be evaluated on this aspect explicitly. More specifically, it is advisable to (1) consider evidence from existing literature that the candidate task will capture the cognitive domain under scrutiny (eg, are there studies relating the VR task to other assessments whose domain specificity is better established?) and (2) to vet the candidate task for potential domain confounds (eg, how strongly are visual attention or motor components implicated in solving the task?).

### Ecological Relevance

VR enables researchers to simulate real-world scenarios while maintaining a high degree of experimental control. Increasing a task’s similarity to the actual challenges encountered by patients in the real world may facilitate diagnostic and rehabilitative approaches that more adequately address the patients’ real-life deficits. This line of thought is commonly subsumed as the potential of VR to increase a task’s *ecological validity* [1,24,32]. As noted above, there is an ongoing debate on what this umbrella term should and should not include on a conceptual level and whether a more fine-grained approach, perhaps along the axes of representativeness and generalizability [26], would be beneficial. In opting for the term *ecological relevance*, we focus on the patient perspective of everyday functional demands. We thereby deliberately scrutinize potential cognitive deficits of a patient in the domain under study that are likely to translate into real-world outcomes, such as the ability to function in the real-life environment and perform a real-life action. A candidate task is thus evaluated based on how closely it reflects these demands as encountered by the study population of interest. In consequence, a judgment is made on how relevant the paradigm is to the user’s everyday life with respect to (1) the virtual environment in which the task is set, (2) the experimental stimuli to which the user is exposed, and (3) the activities performed to solve the task (ie, the user response).

### Technical Feasibility

Although a candidate paradigm may possess a variety of desirable properties, one may encounter technical limitations when implementing the paradigm in VR. Technical feasibility is especially important to consider if the paradigm is designed de novo or if previously computerized versions of an existing task are not available or incompatible with state-of-the-art VR setups. We therefore evaluate whether the task can be sensibly implemented in VR in general and whether the implementation is compatible with a head-mounted display (HMD), with a 2D display device such as a tablet or a desktop computer, or both. Moreover, it is important to assess whether user interaction and navigation in the virtual world require further input devices such as VR controllers or a mouse, and if so, which input devices are technically feasible. Importantly, the technical feasibility of a candidate task may be constrained by project-specific factors such as the necessity of using a particular HMD model, a specific interaction device, or an examination room with spatial limitations.

### User Feasibility

Candidate paradigms must further be evaluated in terms of feasibility for different user groups. First, is the candidate task expected to be feasible in healthy users, also considering potential differences across different age groups? Second, can one expect it to be viable in the patient population of interest, and are there possibilities to alleviate obstacles to maximize patient feasibility? Third, the task is evaluated on the complexity of the user interaction and navigation in the virtual world: How difficult is it to move and act in the virtual environment? How long will it take for healthy users and patients to learn how to carry out the task, and how intuitive are the controls? Furthermore, task duration and attentional demands might limit user feasibility. Therefore, it must be considered how long the task will take on average and whether the target user group can be expected to focus on the task sufficiently. Moreover, user feasibility may be hampered by VR-induced adverse symptoms and effects (VRISE), which are not only important for safety considerations but also because VRISE are likely to confound task performance [33]. One should therefore judge the paradigm on the likelihood of inducing VRISE such as VR-related kinetosis (*cybersickness*). Finally, it is important to evaluate any ethical concerns the task may implicate, such as the presentation of strong fear-inducing stimuli or safety considerations, as mentioned above (see also Madary and Metzinger for a detailed review of ethical considerations in VR [34]). Although these are relevant aspects to evaluate in any study population, the judgment on what is feasible in the target user group may certainly differ depending on population-specific factors such as health status or age.

In practice, the maximization of user feasibility is linked to development principles from human-computer interaction (HCI) and user experience (UX) research. This includes the application of ergonomic principles and human-centered design that maximizes accessibility and involves users and other stakeholders in an iterative development process [35,36]. Concerning virtual worlds, standard UX heuristics remain crucial [37], although some VR-specific components such as sense of control and multimodal interaction warrant additional consideration, as they have been shown to affect UX [38,39] and may be especially important in potential future multiuser scenarios and in users with neurological disorders [40].

### User Motivation

Beyond mere feasibility, user motivation is crucial to ensure that participants will engage in the candidate task, especially in repeated application. To optimize user compliance, it is therefore advisable to evaluate the task with regard to factors that may facilitate user motivation. First, users may be intrinsically motivated to carry out the task due to high expected benefit or face validity of the paradigm. Second, the entertainment factor of the candidate task is judged. Next, one evaluates the possibility of a reward system, both within-session (eg, a virtual reward for successful task completion) and across-session (eg, a high-score system or advancing to higher levels). Furthermore, we examine the possibility of within- and across-session feedback on user performance. These features touch upon a gamification approach to cognitive assessment [41,42], and this represents one aspect in which VR is particularly capable of playing off its strengths against the classical neuropsychological assessments.

### Task Adaptability

The ability to adapt the candidate paradigm carries important implications for both clinical and experimental settings. First, it is useful to consider how easily parallel versions of the candidate task can be created, which represents a major limitation of many classical neuropsychological tests. Not least, this aspect also constitutes a prerequisite for applying the paradigm repeatedly, for instance in a pre- and postintervention study design. Second, the task is judged on how well its difficulty can be (parametrically) adjusted. The required levels of difficulty may vary markedly between study populations (eg, patients vs healthy controls, younger vs older participants) or across multiple sessions in repeated within-participant applications. Therefore, the task is evaluated with respect to experimental parameters that can be effectively manipulated to affect task performance systematically. In addition, it should be considered if task difficulty is adaptable enough to induce sufficient across-participant performance variance and avoid floor and ceiling effects.

### Performance Quantification

A further important prerequisite for a suitable candidate paradigm concerns the ability to measure user performance in a quantitative way. One should therefore consider if outcome variables to quantify performance have been defined, or if they can be derived from the data obtained in VR. As behavior in virtual environments can be tracked digitally with high resolution in both time and space, VR offers increasingly multivariate and experimenter-independent performance parameters, facilitating more objective, data-driven, and automated analysis approaches. It is therefore evaluated to what extent the candidate paradigm allows for experimenter-independent performance evaluation.

Notably, task adaptability and automatic performance quantification in VR complement related advances in contemporary psychometrics. As VR paradigms are centered around user interactions with the virtual environment in real time, they offer the possibility of highly dynamic and individualized testing scenarios, enabling more precise and time-efficient assessments in accordance with the ideas of computerized adaptive testing [43,44]. Implementing such a reactive task design also facilitates the inclusion of large item pools with predefined difficulty of sufficient variance as well as real-time scoring (ie, immediate item evaluation) to automatically utilize the most informative items based on the participant's current performance and the assessment's goal.

### Immersive Capacities

Another dimension not adequately captured by traditional test criteria concerns the capacity of VR systems to create the illusion of being located in the virtual world. There is an ongoing conceptual debate about the technical terms describing this phenomenon, specifically *immersion* and its relation to and disentanglement from the notion of *presence* [45-48]. For paradigm development, we follow Slater in the distinction that immersion describes a VR system’s objective technical properties that support natural sensorimotor contingencies, whereas presence refers to the subjective illusion of *being there* in the environment as a subjective correlate of immersion [49-51]. Accordingly, one first evaluates the degree of immersion as specified by task factors and the VR system necessary to present this task. Second, the likelihood that the task (in its final implementation) will facilitate the illusion of being in the virtual environment is considered, and ideally, this judgment is informed by prior empirical evidence using presence questionnaires. This evaluation is important for two reasons: first, the degree to which participants feel present in the virtual environment may either have direct implications for the research question or represent a latent factor influencing task performance or user engagement, constituting a potential confound. Second, the state-of-the-art VR technology raises hopes that a higher degree of presence could be beneficial in diagnostic assessment, cognitive training outcome, or UX (with respect to the latter, see Brade et al [52] and Lorenz et al [53]). Indeed, there is some evidence that increased presence may have a positive impact on participants’ cognitive performance, for instance, regarding fact learning [54] or memory encoding [55], although potential benefits of increased presence in clinical assessment remain to be explored.

### Training Feasibility

A further consideration concerns the feasibility of the candidate paradigm to serve as the basis for a training tool. In a one-time application setting (eg, purely diagnostic assessment), the paradigm needs to fulfill fewer requirements compared with a repeated-application setting (eg, implementation of a cognitive training tool). First, one should evaluate whether there are any practical obstacles to the repeated application of the paradigm. This concerns the logistics of the VR system used for the task: Can the task be administered in multiple sites or at home, or must the user be tested in a specialized laboratory, for instance, due to the necessity of specific interaction devices such as a VR treadmill or a cave automatic virtual environment (CAVE) [56]? Potential caveats in user feasibility may yield cumulative disadvantages in the training scenario (eg, mild risk of cybersickness may be acceptable in a one-time application but could decrease compliance when repeated with high frequency). Second, one determines if the necessary prerequisites of task adaptability are met (eg, the possibility to create parallel versions, effective manipulation of difficulty). Third, it is important to consider to what extent the paradigm offers the possibility of conveying cognitive strategies for compensatory training and how these could be implemented (eg, by leveraging the extensive cueing possibilities in VR [1]). Furthermore, the likelihood of transfer effects is examined and if there is any empirical indication of their expected quality regarding near vs far transfer.

### Predictable Pitfalls

Furthermore, it is advisable to vet the candidate paradigm for predictable pitfalls. As in any clinical study, implementing a VR paradigm for cognitive assessment requires time, know-how, and monetary resources that must be weighed against potential knowledge gains and patient benefit. To optimize the potential of the research endeavor, one first evaluates how well the candidate paradigm adheres to the task requirements of the current research project and if the paradigm can be modified to maximize this adherence. Second, it is considered to what extent the application of the candidate paradigm constitutes a reasonable allocation of the study resources. Not least, scrutinizing potential pitfalls early on in the development process also serves as a quality check when designing a VR paradigm de novo.

## Application of the VR-Check Framework

**Figure 2:**
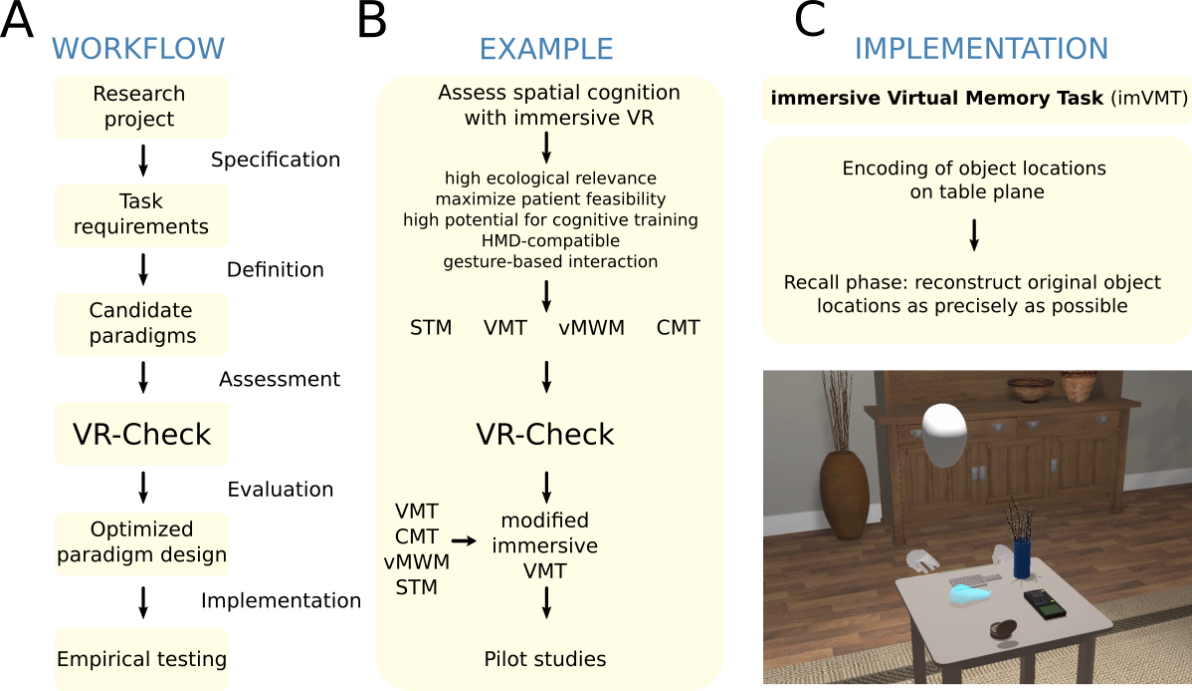
Workflow of the VR-Check evaluation. Panel A summarizes the main general steps for paradigm optimization with the virtual reality check framework. Panel B shows how this workflow applies to an exemplary research project on the assessment of spatial cognition with immersive VR (see main text). Four candidate tasks were evaluated: the Starmaze (STM), the Virtual Memory Task (VMT), the Virtual Morris Water Maze (vMWM), and the Cognitive Map Task (CMT). See Figure 3 and the main text for details on the VR-Check evaluation. Panel C visualizes the result of the optimization procedure: the immersive Virtual Memory Task (imVMT). The screenshot displays a third-person view of a user memorizing the locations of everyday objects on the table. HMD: head-mounted display; VR: virtual reality.

**Figure 3:**
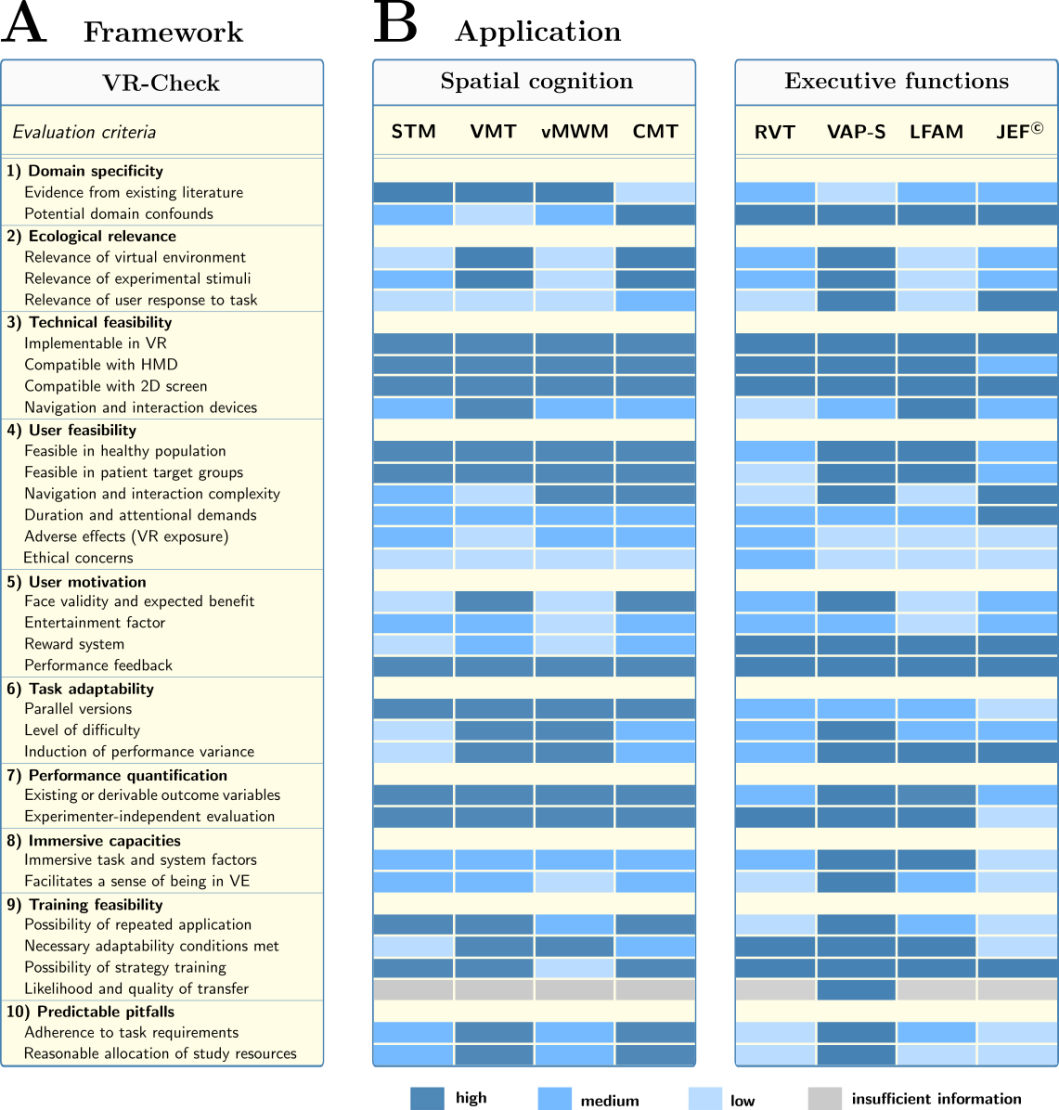
The VR-Check framework for virtual reality paradigms in neuropsychology. Panel A summarizes the evaluation dimensions in the form of a checklist. Panel B visualizes the application of the framework for the exemplary cases of assessing spatial cognition and executive functions with immersive VR. The color schemes display the item-wise consensus ratings on the degree to which the feature is fulfilled for a given paradigm. The evaluation procedure is illustrated for the Starmaze (STM), Virtual Memory Task (VMT), the Virtual Morris Water Maze (vMWM), and the Cognitive Map Test (CMT) for assessing spatial abilities, and the Ride in a Virtual Town (RVT), the Virtual Action Planning-Supermarket (VAP-S), the Look For A Match (LFAM) task as well as the Jansari assessment of Executive Functions (JEF) for assessing executive functions. For the given task requirements, the VMT, CMT, and the VAP-S emerged as the most suitable paradigms for the development of an immersive VR application, as detailed in the main text. 2D: 2-dimensional; HMD: head-mounted display; VE: virtual environment; VR: virtual reality.

The following sections illustrate how systematic evaluation with the VR-Check framework can guide the decision-making process in defining a neuropsychological VR paradigm for a specific research project (see Figures 2 and 3).

### Evaluation Workflow

First, the properties required of the VR paradigm are defined. Of note, these task requirements are necessarily project specific, such that the relative weight of the various evaluation dimensions will naturally differ across projects. Furthermore, to facilitate a comparative evaluation of tasks across the VR-Check features, a semiquantitative rating is applied to evaluate if a particular feature applies to the candidate task to a high, medium, or low degree, or if there is insufficient information to make an informed judgment (eg, asking *How high is the degree of ecological relevance of the virtual environment in paradigm X to the study population of interest?*). Researchers are thus able to systematically go through the list of features and judge each candidate paradigm according to the description above. For the de novo development of VR paradigms, the same process is applied to competing ideas or prototypes, yielding an explicit account of which task features need to be maximized.

### Example Project: Task Requirements and Candidate Paradigms

Here, the results of this evaluation procedure are presented for an exemplary research project emanating from our consortium. It is important to note that the following outcomes do not represent a judgment on the value of the paradigms *per se*, but the outcomes rather provide an illustration of the evaluation process itself and how it can inform project-specific paradigm optimization.

The goal of the exemplary research project was to apply immersive VR for the neuropsychological assessment of spatial cognition and executive functions. Suitable tasks were required to (1) be relevant to participants’ everyday life, (2) be feasible in a wide range of neurological patient populations, (3) inform the development of a subsequent cognitive training tool, (4) be implemented with an HMD, and (5) allow for natural user interaction based on body-tracking devices and gesture recognition. The technical setup for implementing the task included an Oculus Rift headset (Oculus, Facebook Technologies, LLC, Menlo Park, CA), a Leap Motion controller (Leap Motion, Inc, San Francisco, CA) for hand-tracking, and a Microsoft Kinect sensor (for Windows; Microsoft Corporation, One Microsoft Way, Redmond, WA) to support body tracking. Software implementation rested on the Unity game engine (version 2019.2.11f1; Unity Technologies, San Francisco, CA) and Blender graphics suite (version 2.79; Blender, Amsterdam, the Netherlands) for VR development, a custom-built communication middleware, and a custom Web interface for data management.

Candidate paradigms were identified by literature screening of existing VR tasks and in-house paradigms from January to May 2018. All candidate tasks were assessed along the VR-Check dimensions by an interdisciplinary research consortium, including 3 cognitive neuroscientists, 2 physicians, and 4 clinical neuropsychologists. None of the team members was involved in the creation of the considered tasks or had any conflict of interest. Each paradigm was presented to the group by varying team members, followed by subsequent rating. If missing information or divergences in the individual ratings were identified, these issues were addressed in the subsequent session. A consensus was reached on all ratings through group discussion. Potential ties were to be resolved by the senior scientists, although no ties occurred for the considered paradigms. Figure 3 visualizes the ratings for a subset of four promising candidate tasks in each cognitive domain. The description below is limited to a brief account of the most decisive aspects; interested readers are referred to Multimedia Appendix 1 for a detailed point-by-point description of the systematic evaluation.

### Example Project: Spatial Cognition

With respect to spatial cognition, the evaluation process is illustrated in the following candidate tasks: (1) the *Starmaze* (STM) [57-59], a VR adaptation of a rodent paradigm [60] to differentiate egocentric from allocentric navigation strategies, in which the user navigates through a point-symmetric star-shaped labyrinth to find a target; (2) the *Virtual Memory Task* (VMT) [32], a computerized spatial memory task similar to an existing real-life task [61], in which participants are required to memorize locations of everyday objects on a table; (3) the *Virtual Morris Water Maze* (vMWM) [62,63], a VR adaptation of the classical place navigation task originating from rodent research [64], in which participants learn to navigate to a concealed platform; and (4) the *Cognitive Map Task* (CMT) [65,66], a spatial learning paradigm in which participants have to construe, maintain, and retrieve a cognitive map of a virtual town by learning and finding landmarks.

On the basis of the assessment along the VR-Check dimensions, the STM and the vMWM although certainly highly appropriate paradigms for other research questions were judged to be less favorable for our purposes due to limited ecological relevance, task adaptability, and training potential. In contrast, the VMT emerged as the paradigm that most closely adhered to our task requirements, made explicit through point-by-point assessment along the VR-Check dimensions: besides high ecological relevance to our target population, favorable user feasibility, and excellent adaptability, it avoids some of the caveats of other candidate paradigms (such as high navigation complexity or the risk of adverse effects) and demands comparatively moderate implementation efforts, rendering it the optimal allocation of our study resources. Nonetheless, the VMT is limited to an assessment of spatial memory capacities due to the comparatively narrow domain target. In terms of assessing navigational abilities, the CMT was evaluated to be the most suitable starting point for the development of an immersive paradigm because of favorable ecological relevance, user feasibility and motivation, and high training potential. Notwithstanding, our evaluation process also identified potential improvements of the CMT that have to be addressed in the development process, such as a more fine-grained adaptation of difficulty.

### Example Project: Executive Functions

*Executive functions* is an umbrella term for a multifaceted construct, including several interconnected high-level cognitive abilities that serve ongoing, goal-directed actions [67]. Subdomains include planning, problem solving, monitoring, working memory, inhibition, and task switching, and despite ongoing terminological disambiguations, there is relative agreement on the complexity and superordinate coordination role of executive functions and their importance regarding human adaptive behavior [67-70]. For a comprehensive review of executive functions paradigms in VR, see Parsons [25] and Valladares-Rodríguez et al [71]. As mentioned above, we exemplify evaluation outcomes in 4 candidate paradigms: (1)* a Ride in a Virtual Town* (RVT) [72], a prospective memory task featuring a car drive using real car components as interaction devices while completing a list of errands; (2) the *Virtual Action Planning-Supermarket* (VAP-S) [31], a grocery shopping task; (3) the *Look For A Match* (LFAM) task [73], an adaptation of the Wisconsin Card Sorting Task to a virtual beach environment; (4) the *Jansari assessment of Executive Functions* (JEF) [74], a multistep office task requiring multitasking to prepare a meeting on time.

Resulting from the VR-Check evaluation, some inconsistencies with our task requirements were identified for the LFAM (limited ecological relevance to our target populations, drawbacks in user motivation), the RVT (risk of adverse effects, incompatibilities with our interaction requirements, ecological relevance limited to drivers, ethical concerns about loss of driving capability in patient population, limited training feasibility), as well as the JEF (user feasibility limited to higher-functioning populations, ecological relevance restricted to a subgroup of our target population, incompatibilities with our immersive system factors, limited training feasibility due to caveats in task adaptability). The VAP-S, in contrast, was evaluated to be highly consistent with the project’s task requirements regarding user feasibility, technical requirements, ecological relevance, and training potential, while demanding reasonable implementation efforts. The VAP-S was therefore esteemed the most favorable basis for the development of an immersive executive functions paradigm. Nonetheless, the systematic evaluation also highlighted potential caveats of the paradigm (limited domain specificity, technical solution required for large-scale multidirectional locomotion), which can thus be explicitly optimized in the implementation process.

## Discussion

To leverage the potential of VR in neuropsychology, researchers are increasingly challenged with optimizing the experimental paradigm to address the study question at hand. The body of literature on biomedical VR applications is growing fast, and the importance of cognitive research within this field is steadily increasing (Figure 1), supported by the increasing availability of VR hardware and software systems. With these developments, the need arises for a new methodological framework on systematic paradigm evaluation. This gap is aggravated further by the inability of the traditional quality criteria to capture the multifaceted nature of contemporary VR. With this work, we aim to address this gap with a multidimensional evaluation protocol for VR applications in neuropsychology, summarized as an easy-to-use checklist (VR-Check, Figure 3).

### Paradigm Optimization and Across-Domain Trade-Offs

The systematic evaluation approach of the VR-Check framework raises the general question of what constitutes an ideal VR paradigm for neuropsychological research. Surely, if we defined an entertaining, highly adaptable, easy-to-play, easy-to-implement, highly immersive task that is viable for any user group, targets a well-circumscribed cognitive domain, adequately captures cognitive deficits relevant to everyday functioning as measured objectively by experimenter-independent performance outcomes, and which can be applied repeatedly to induce systematic improvement in both the tested and further cognitive domains, such a paradigm would be welcomed by researchers and clinicians alike.

However, as a corollary of the multidimensional nature of VR, such an endeavor is unrealistic for two principled reasons: first, what is desired of the task is tightly linked to the research question of interest. In consequence, there is no general profile of objectively desirable properties. Although minimal requirements regarding user feasibility or technical implementation must be met by any clinical paradigm, the relative importance of the various domains will differ markedly over research applications and target populations. Indeed, the VR-Check framework serves precisely the purpose of prioritizing which domains are more important than others to address a given research question. This flexibility toward the study purpose enables researchers to weigh the different dimensions against each other and maximize the adherence to their project-specific requirements.

Second, the VR-Check framework illustrates a qualitative difference with respect to the interaction among evaluation criteria in that some are logically congruous, whereas others imply reciprocal incongruities. For instance, a paradigm featuring high training feasibility must also fulfill a variety of requirements concerning technical feasibility, user feasibility, and task adaptability and is more likely to be judged favorably in terms of user motivation because these dimensions, to some extent, inform the evaluation of training feasibility. In contrast, other comparisons yield across-domain trade-offs. Specifically, this concerns the relationship between cognitive domain specificity and ecological relevance. In the attempt to target a specific cognitive domain with high precision, the recruitment of other cognitive domains must be minimized. However, this is rarely the case in everyday functioning, when a multitude of cognitive domains are engaged simultaneously. A VR paradigm featuring high ecological relevance will therefore necessarily concede some domain specificity by recruiting other domains than the one intended. Inversely, a VR paradigm featuring high domain specificity permits only limited relevance to cognitive functioning in real life because of an artificially narrow cognitive target. As a result of this incongruousness, a deliberate decision must be made on the trade-off between domain specificity and ecological relevance.

A similar point arises with respect to the relationship between ecological relevance and experimental control. Although both task and environment are highly controllable in VR, the increased degrees of behavioral freedom can result in less controlled participant behavior as compared with classical neuropsychological assessments. This behavioral freedom comes with an increased number of error sources not related to the cognitive task itself, such as visual attention, motor control, or navigational demands. In the research context, we can increase experimental control by restricting what the participant can and cannot do in VR. However, this again entails decreased relevance to everyday functioning, as real-life behavior offers similarly many degrees of freedom and also encompasses a multitude of error sources.

In summary, the properties required of a VR paradigm are dependent on the research question at hand, and there are inevitable across-dimension trade-offs in paradigm design. These aspects necessitate deliberate design decisions to permit the project-specific optimization of the VR paradigm. The VR-Check framework guides this optimization process because it allows for a systematic account of how well a paradigm adheres to the project-specific requirements and because it makes these design decisions explicit.

### Toward Improved Standardization of Clinical Virtual Reality Applications

Although the assets of VR for clinical research have been examined before, previous approaches have predominantly addressed general favorable properties of the technology [1-3,5,6] or focused on specific aspects of VR application such as avoiding VR-related adverse effects [33,75], improving UX [36,38] or ethical adversities [34]. Other studies have suggested design considerations derived from specific VR applications [76], focused on rehabilitative tools [77], or dealt with clinical study design for VR-based therapies [78].

The VR-Check framework complements these studies, as it specifically targets the project-specific optimization of the paradigm (rather than the study) design and explicitly addresses cognitive and behavioral research, and because it provides researchers with a general and easy-to-use evaluation tool. However, even though the application of the framework was highly informative in the exemplary research project, some limitations of this work deserve mentioning. First, the application of the framework was limited to the assessment of spatial cognition and executive functions, such that further research is necessary to corroborate its utility with respect to other research questions. Moreover, current evaluation outcomes are limited to semiquantitative assessment and consensus ratings, warranting further work to solidify more quantitative approaches and assess the rates of agreement across individual raters. Furthermore, it should be noted that paradigms that have been applied more often in literature might lead researchers to evaluate them more favorably simply because existing evidence makes these paradigms easier to judge. However, the fact that a paradigm may be more established in the literature does not necessarily imply that it is better suited for the study question at hand. Finally, we focused here on the design optimization of VR paradigms for neuropsychological assessment. Nonetheless, the value of therapeutic VR applications is becoming increasingly apparent [78-80], and there is an important interplay between assessment and rehabilitation, especially with regard to devising individualized therapies that cater to the patient’s specific deficits (*precision medicine*). Although many of the VR-Check dimensions appear relevant to clinical VR tasks in general (eg, technical and user feasibility, adaptability, or outcome quantification), future work must investigate if the protocol is also applicable to VR tools for cognitive training and rehabilitation, or to what extent the framework must be modified to enable paradigm optimization for these applications.

Even with these limitations in mind, the VR-Check framework represents a first step toward the standardized optimization of VR paradigms in clinical neuropsychology. The potential of contemporary VR is contrasted by a relative scarcity of consensus on what should be regarded as best practice when applying VR in clinical research. With respect to paradigm development, the VR-Check framework can inform this discussion. Even with optimal paradigm design, however, proof of clinical utility ultimately requires high-quality empirical evidence such as randomized controlled trials (RCTs). In this context, the newly established Virtual Reality Committee of Outcomes Research Experts (VR-CORE) has recently suggested a framework for the development and validation of VR-based therapies [78]. This framework features 3 study phases (VR1-VR3) similar to the phase I-III model of pharmacological intervention studies. Although the authors’ approach focuses explicitly on VR treatments, their systematic methodological framework is similar in spirit to our suggestions, and the two approaches complement each other (paradigm design optimization and study design optimization). For instance, the authors’ call for human-centered design in early VR treatment study phases (VR1) is matched by our explicit focus on the patient perspective in the domains of technical feasibility, user feasibility, user motivation, and ecological relevance. The intermediate trial phase (VR2) is concerned with initial feasibility testing and can thus be regarded as the *study design* counterpart to the *paradigm design* feasibility dimensions of the VR-Check framework. The later VR-CORE phase (VR3) concerns RCTs to examine VR treatment efficacy vs a control condition. As such, the extension of the VR3 phase to neuropsychological assessment tasks seems natural: where a VR treatment must show intervention efficacy, a VR assessment task must show discriminatory or predictive power in empirical evaluation.

As methodological guidelines such as the VR-CORE recommendations and the VR-Check framework are further developed, they may ultimately synergize in pursuit of a more rigorous, systematic, and well-informed protocol for the development of clinical VR applications.

## Appendix

Multimedia Appendix 1 Detailed description of the VR-Check application.

## Abbreviations

CMT: Cognitive Map Task
HCI: human-computer interaction
HMD: head-mounted display
JEF: Jansari Assessment of Executive Functions
LFAM: Look for a Match
MET: Multiple Errands Test
RCT: randomized controlled trial
RVT: Ride in a Virtual Town
STM: Starmaze
UX: user experience
VAP-S: Virtual Action Planning-Supermarket
VE: virtual environment
VMT: Virtual Memory Task
vMWM: Virtual Morris Water Maze
VR: virtual reality
VRISE: VR-induced adverse symptoms and effects
