# Supplementary material for: Multidimensional Evaluation of Virtual Reality Paradigms in Clinical Neuropsychology: Application of the VR-Check Framework
Source: J Med Internet Res. 2020 Apr 27;22(4):e16724. doi: 10.2196/16724 (PMC7215516; doi:10.2196/16724)
Supplement: Multimedia Appendix 1 [file jmir_v22i4e16724_app1.docx]

## Appendix

The following provides a detailed description of the evaluation process illustrated in the main text, based on a systematic point-by-point application of the VR-Check dimensions.

### Example project: spatial cognition

Evaluations concerns the *Starmaze* (STM) [1-3], the *Virtual Memory Task* (VMT) [4], the *Virtual Morris Water Maze* (vMWM) [5, 6], and the *Cognitive Map Task* (CMT) [7, 8], as detailed in the main text.

Domain specificity was rated high for the STM and vMWM (spatial memory and navigation), with moderate domain confounds of selective attention (STM) and executive capabilities (vMWM) [6]. The VMT was also evaluated to feature high domain specificity, as it closely targets visuospatial memory (without navigational demands) and showed congruency with construct-driven tests in prior empirical evaluation [4], while potential domain confounds were judged to be comparatively low (moderate short-term memory capacity and low motor demands). The CMT was evaluated to yield low domain specificity and high potential for domain confounds, as the task is dependent on strategy learning and action planning besides spatial memory and navigation. Ecological relevance of the virtual environment was rated low for the STM and the vMWM due to the labyrinth setting, while the VMT (house setting) and the CMT (town setting) were rated high in this aspect. Ecological relevance of the experimental stimuli was deemed high for the VMT (household objects) and the CMT (streets and shops), moderate for the STM (artificial maze but with natural landmarks such as mountains), and low for the vMWM (artificial pool). In contrast, ecological relevance of the user response to solve the task was considered low for the STM and vMWM (finding a target in a maze) as well as the VMT (relocating objects after removal) and moderate for the CMT (building a cognitive map for orientation). All tasks were regarded as highly VR-feasible since computerized versions already exist. However, technical feasibility with respect to moving in the virtual environment was deemed moderate for the STM, vMWM, and CMT, as all feature large-scale locomotion (or alternatively swimming for the vMWM), increasing the difficulty of technical implementation with respect to tracking-based navigation. In this regard, feasibility was judged high for the VMT (hand-object interaction, low interaction complexity, no large-scale locomotion required). Feasibility in both healthy and patient populations were rated high for all of the examined paradigms [4, 8-12]. Navigation complexity was considered high for the CMT and vMWM (unrestricted multidirectional motion), moderate for the STM (multidirectional motion but restricted by maze walls), and low for the VMT (no large-scale motion). While adverse effects were not a problem for any of the tasks in the 2D version, the potential for VR-induced kinetosis was deemed moderate for the STM, vMWM, and CMT due to the necessity of large-scale motion in the immersive setting, while the risk of adverse effects was judged to be low for the VMT. All paradigms were judged to be ethically innocuous under the condition that potential adverse effects could be kept to a minimum. In this context, it is noteworthy that König an colleagues reported a few cases of negative emotional reactions when patients suddenly realized deficits in spatial memory upon conducting the 2D VMT [4].

Regarding user motivation, expected benefit was considered to be low for the STM and vMWM due to the artificial nature of the tasks, while it was deemed high for the VTM and CMT, as our target study population would likely be able to relate to the purpose of the assessment. The entertainment factor was judged moderate for the STM, VMT, and CMT in their current versions and low for the vMWM due to a comparatively higher degree of repetitiveness. Feasibility of a reward system was deemed low in the STM (as the task’s focus is to disentangle navigational strategies rather than assess navigational abilities) and the vMWM (due to the repetitive character) and moderate for the VMT (e.g., new or surprise objects) and CMT (e.g., access a new part of town). The possibility of performance feedback was considered high for all tasks. With respect to task adaptability, the feasibility of creating parallel versions was judged to be high for all tasks. The possibility of effectively manipulating difficulty levels, however, was judged to be high for the vMWM (e.g., size of pool, size of platform) and the VMT (e.g., number of memory items, encoding intervals), moderate for the STM (e.g., number of landmarks or more alleys, but this could induce navigation strategy confounds), and low for the CMT (e.g., size of town or complexity of cognitive map, but this could increase executive demands, aggravating domain confounds). As regards the induction of performance variance, the STM has shown ceiling effects in young healthy participants and floor effects in patients with Alzheimer’s Disease and Mild Cognitive Impairment [12]. For the CMT, while there was some indication of ceiling effects in young healthy adults [7], it also induced variance with age [8]. In contrast, performance variability was evaluated high for the vMWM [10, 13] as well as the VMT [4].

With respect to performance quantification, objective outcome measures were abundant for the STM (e.g., number of correct trials, path lengths, trial times), the VMT (e.g., distance errors, accuracy by memory items, placement analysis, trial times), the vMWM (e.g., latency and distance to platform, path complexity, duration), and the CMT (e.g., mean time delay from optimal route, detour errors) alike. Experimenter-independent automated quantification of these measures was considered easily possible for all paradigms. All tasks were judged to be compatible with our immersive system requirements, although moderate difficulties were anticipated in implementing locomotion (STM, vMWM, and CMT) and hand-object interaction (VMT), respectively. As gesture-based interaction was one of the requirements, the capacity to facilitate a sense of being in the virtual environment was considered medium for the STM (locomotion), VMT (object manipulation), and CMT (locomotion), and low for the vMWM (swimming). With regard to training feasibility, no general obstacles to repeated application were identified for any of the tasks, although some moderate drawbacks were identified for the vMWM due to the limited user motivation. While the STM was evaluated to show high potential for strategy training (as the paradigm disentangles between navigational strategies with high precision), there were concerns in how adaptable the task would be for training, which was also the case for the CMT, but to a lesser extent. The VMT was judged to fulfill both these properties to a high extent, while the vMWM was considered to possess low potential for strategy training. Likelihood of transfer was difficult to judge here, as none of the examined tasks has been developed into a training paradigm yet.

Inconsistencies with the defined task requirements were identified for the STM and the vMWM due to limited ecological relevance, task adaptability, and training potential. In contrast, the VMT closely adhered to our task requirements based on high ecological relevance to our target populations, favorable user feasibility and excellent adaptability, while the CMT was evaluated to be the most suitable starting point for the development of an immserive navigation paradigm because of favorable ecological relevance, user feasibility and motivation, and high training potential.

### Example project: executive functions

Evaluation outcomes are provided for *A Ride in a Virtual Town* (RVT) [14], the *Virtual Action Planning- Supermarket* (VAP-S) [15], the *Look for a Match* task (LFAM) [16], and the *Jansari Assessment of Executive Functions* (JEF©) [17], as outlined in the main text.

Regarding domain specificity, we found that the RVT, LFAM and JEF© all focus on moderately narrow executive subdomains, such as prospective memory or mental flexibility. In contrast, the VAP-S lists several subdomains, all subserving the activity of daily living “shopping,” rendering the targeted domain spectrum comparatively wider. As mentioned above, the nature of executive functions lies in their superordinate functions, such that overlap with subordinate cognitive domains such as attention or memory is to some extent inevitable. Accordingly, all four tasks were considered to feature high potential for domain confounds. The four paradigms were evaluated to show considerable differences in their ecological relevance to our target population: Shopping in a supermarket is a frequent everyday activity for most people including the elderly and many neurological patients, such that the VAP-S was considered to be highly ecologically relevant in terms of the environment, stimuli, and user response to the task [18]. In comparison, the ecological relevance of the environment and the stimuli in the RVT is restricted to drivers, and the user response to the task contains some artificial elements (e.g., oral execution of errands, town with only one street). Similarly, the ecological relevance of the environment and the experimental stimuli in the JEF© (office workspace) is limited to white-collar workers, although the user response to the task (multitasking to meet a temporal deadline) was considered to be of high ecological relevance to the target population. In contrast, ecological relevance was rated low in the LFAM for the environment (beach bar), stimuli (set of umbrellas), and user response (find out the hidden rules about which customer has ordered which product). All tasks were evaluated to be feasible for 2D implementation, but there were concerns about the HMD compatibility of the JEF© due to the dependence on hard copy material for execution. Compatibility with our technical interaction requirements (body-tracking and gesture recognition) was deemed high for the LFAM (hand-object interaction), medium for the JEF© and VAP-S (large-scale movement as locomotion), and low for the RVT (large-scale movement in car while seated). Interaction complexity, on the other hand, was low for the RVT (unidirectional movement, oral commands) and the LFAM (no locomotion, manual interaction), while it was considered high for the VAP-S and JEF© (multidirectional large-scale motion and object interaction). While the LFAM and VAP-S were considered highly feasible in both healthy and patient target populations (for the VAP-S, see Josman et al. [19, 20]), the JEF© was judged feasible only in higher-functioning populations (although alternative scenarios to overcome this limitation are being developed [21]). Similarly, the RVT was considered to show moderate and low feasibility in the target healthy and patient groups, respectively, due to the driving setting. Moreover, among the considered paradigms, the RVT is the only one to report significant drop-outs due to cybersickness (15% in an older population [14]). Furthermore, as a non-negligible amount of our patient group could be expected to have lost their license or capacities to drive, we judged that confronting patients with this often highly emotional topic raises ethical concerns. Attentional demands were deemed high for the JEF© (due to the higher multitasking load and temporal density) and moderate for the remaining tasks.

The expected benefit of the LFAM was evaluated to be low in our target populations due to the low ecological relevance, and similarly for the JEF©, as the majority of our patients would not be expected to relate to the office setting. The expected benefit of the RVT was considered medium (based on the errands-running part), while the VAP-S scored high due to the very common task scenario. The entertainment factor was deemed low for the LFAM due to the repetitive character (although it does compare well against the original WCST), and otherwise considered moderate. The VAP-S, however, is the only paradigm for which explicit patient-reported outcomes are available, showing medium to high enjoyment and satisfaction. All tasks were evaluated to lend themselves well to reward systems and performance feedback. Concerning the creation of parallel versions, implementation demand differs among the paradigms. For example, producing a parallel large-scale town (RVT) requires higher VR modeling resources than rearranging shelves and products in a supermarket (VAP-S). Nonetheless, for both tasks, creating parallel versions based on varying errand lists poses comparably little implementation effort (and a similar adaptation would be possible for the LFAM). In contrast, creating parallel tasks for each of the nine subdomains of the JEF© would require considerably more development resources, which was considered less feasible for our purposes. The grading of difficulty was deemed to be easy for the VAP-S (e.g., size of supermarket, complexity of shopping list), while the narrow task demands (LFAM), the reduction to list-solving (RVT; an action is performed by saying the action), and the implementational challenges (JEF©) were judged to pose moderate obstacles to difficulty grading for the other paradigms, respectively. Induction of across-participant variance has been shown for all tasks, with the limitation of moderate ceiling effects in healthy young participants for the RVT. In terms of performance quantification, the VAP-S and LFAM both offer a range of time- and accuracy-based outcome variables, whereas the RVT and the JEF© in their current forms report a comparatively limited number of (discretized) outcomes. The JEF© requires time-consuming and non-automatized post-hoc ratings of the performance, limiting the feasibility of experimenter-independent evaluation (although inter-rater reliability was high in a small pilot study), while this was esteemed well feasible for the remaining tasks.

As regards immersive capacities, compatibility issues with the required system factors were identified for the JEF© (HMD feasibility) and the RVT (driving-wheel interface but oral actions), while the VAP-S and LFAM both lend themselves well to the gesture-recognition and tracking prerequisites. Likelihood of presence was esteemed low for the JEF© (limited to 2D presentation) and RVT (restricted natural interaction) and moderate for the LFAM (artificial beach environment in current form), while this aspect has been empirically assessed for the VAP-S, yielding good presence ratings [22]. With respect to training potential, repeated application was considered well feasible for the VAP-S, but limited for the RVT (risk of cybersickness), the LFAM (drawbacks in user motivation due to repetitiveness), and the JEF© (resource-demanding test setting). Furthermore, some concerns about task adaptability were raised regarding the latter (lack of parallel versions), even though implementing strategy cues was deemed well feasible for all tasks. However, the VAP-S is the only paradigm that has already been used as a training task in a pilot study [23], with promising results concerning training transfer.

Inconsistencies with our task requirements were identified for the LFAM (limited ecological relevance to our target populations, drawbacks in user motivation), the RVT (risk of adverse effects, incompatibilities with our interaction requirements, ecological relevance limited to drivers, ethical concerns about loss of driving capability in patient population, limited training feasibility) as well as the JEF© (user feasibility limited to higher-functioning population, ecological relevance restricted to a subgroup of our target population, incompatibilities with our immersive system factors, limited training feasibility due to caveats in task adaptability). The VAP-S, in contrast, was evaluated to be highly consistent with the project’s task requirements regarding user feasibility, technical requirements, ecological relevance, and training potential, while demanding reasonable implementation efforts.

### Abbreviations

CMT: Cognitive Map Task

HMD: head-mounted display

JEF©: Jansari Assessment of Executive Functions

LFAM: Look for a Match

MET: Multiple Errands Test

RCT: randomized controlled trials

RVT: Ride in a Virtual Town

STM: Starmaze

VAP-S: Virtual Action Planning–Supermarket

VE: virtual environment

VMT: Virtual Memory Task

vMWM: Virtual Morris Water Maze

VR: Virtual Reality

## **References**

1. Iglói K, Zaoui M, Berthoz A, Rondi-Reig L. Sequential egocentric strategy is acquired as early as allocentric strategy: Parallel acquisition of these two navigation strategies. Hippocampus 2009;19:1199-1211. doi:10.1002/hipo.20595

2. Iglói K, Doeller CF, Berthoz A, Rondi-Reig L, Burgess N. Lateralized human hippocampal activity predicts navigation based on sequence or place memory. Proceedings of the National Academy of Sciences 2010;107:14466-14471. doi:10.1073/pnas.1004243107

3. Iglói K, Doeller CF, Paradis AL, Benchenane K, Berthoz A, Burgess N, Rondi-Reig L. Interaction between hippocampus and cerebellum crus I in sequence-based but not place-based navigation. Cerebral Cortex 2014;25:4146-4154. doi:10.1093/cercor/bhu132

4. Koenig S, Crucian G P, Dünser A, Bartneck C, Dalrymple-Alford JC. Validity evaluation of a spatial memory task in virtual environments. Int J Des Innov Res 2011;6:1-13.

5. Nedelska Z, Andel R, Laczó J, Vlcek K, Horinek D, Lisy J, Sheardova K, Bureš J, Hort J. Spatial navigation impairment is proportional to right hippocampal volume. Proceedings of the National Academy of Sciences 2012;109:2590-2594. doi:10.1073/pnas.1121588109

6. Korthauer L, Nowak N, Frahmand M, Driscoll I. Cognitive correlates of spatial navigation: associations between executive functioning and the virtual Morris Water Task. Behavioural brain research 2017;317:470-478. doi:10.1016/j.bbr.2016.10.007

7. Iaria G, Chen JK, Guariglia C, Ptito A, Petrides M. Retrosplenial and hippocampal brain regions in human navigation: complementary functional contributions to the formation and use of cognitive maps. European Journal of Neuroscience 2007;25:890-899. doi:10.1111/j.1460-9568.2007.05371.x

8. Iaria G, Palermo L, Committeri G, Barton JJ. Age differences in the formation and use of cognitive maps. Behavioural brain research 2009;196:187-191. doi:10.1016/j.bbr.2008.08.040

9. Astur RS, Taylor LB, Mamelak AN, Philpott L, Sutherland RJ. Humans with hippocampus damage display severe spatial memory impairments in a virtual Morris water task. Behavioural brain research 2002;132:77-84. doi:10.1016/s0166-4328(01)00399-0

10. Astur RS, Tropp J, Sava S, Constable RT, Markus EJ. Sex differences and correlations in a virtual Morris water task, a virtual radial arm maze, and mental rotation. Behavioural brain research 2004; 151: 103-115. doi:10.1016/j.bbr.2003.08.024

11. Liu I, Levy RM, Barton, JJ, Iaria G. Age and gender differences in various topographical orientation strategies. Brain research 2011;1410:112-119.

12. Bellassen V, Iglói K, de Souza LC, Dubois B, Rondi-Reig L. Temporal order memory assessed during spatiotemporal navigation as a behavioral cognitive marker for differential Alzheimer's disease diagnosis. Journal of Neuroscience 2012;32:1942-1952. doi:10.1523/JNEUROSCI.4556-11.2012

13. Moffat SD, Resnick SM. Effects of age on virtual environment place navigation and allocentric cognitive mapping. Behavioral neuroscience 2002;116:851. doi:10.1037/0735-7044.116.5.851

14. Lecouvey G, Gonneaud J, Piolino P, Madeleine S, Orriols E, Fleury P, Eustache F, Desgranges B. Is binding decline the main source of the ageing effect on prospective memory? A ride in a virtual town. Socioaffective neuroscience & psychology 2017;7:1304610. doi:10.1080/20009011.2017.1304610

15. Klinger E, Chemin I, Lebreton S, Marié RM. Virtual action planning in Parkinson's disease: A Control study. Cyberpsychology & Behavior 2006;9:342-347. doi:10.1089/cpb.2006.9.342

16. Elkind J. S, Rubin E, Rosenthal S, Skoff B, Prather P. A simulated reality scenario compared with the computerized Wisconsin Card Sorting Test: An analysis of preliminary results. CyberPsychology & Behavior 2001;4:489-496. doi:10.1089/109493101750527042

17. Jansari AS, Devlin A, Agnew R, Akesson K, Murphy L, Leadbetter T. Ecological assessment of executive functions: a new virtual reality paradigm. Brain Impairment 2014;15:71-87. doi:10.1017/BrImp.2014.14

18. Aubin G, Béliveau MF, Klinger E. An exploration of the ecological validity of the Virtual Action Planning-Supermarket (VAP-S) with people with schizophrenia. Neuropsychological rehabilitation 2018;28:689-708. doi: 10.1080/09602011.2015.1074083

19. Josman N, Klinger E, Kizony R. Performance within the virtual action planning supermarket (VAP-S): an executive function profile of three different populations suffering from deficits in the central nervous system. Maia Portugal: Proc 7th ICDVRAT 2008.

20. Josman N, Kizony R, Hof E, Goldenberg K, Weiss PL, Klinger E. Using the virtual action planning-supermarket for evaluating executive functions in people with stroke. Journal of Stroke and Cerebrovascular Diseases 2014;23:879-887. doi: https://doi.org/10.1016/j.jstrokecerebrovasdis.2013.07.013

21. Gilboa Y, Jansari A, Kerrouche B, Uçak E, Tiberghien A, Benkhaled O, Aligon D, Mariller A, Verdier V, Mintegui A, Abada G. Assessment of executive functions in children and adolescents with acquired brain injury (ABI) using a novel complex multi-tasking computerised task: The Jansari assessment of Executive Functions for Children (JEF©). Neuropsychological rehabilitation 2017;1-24. doi: https://doi.org/10.1080/09602011.2017.1411819

22. Werner P, Rabinowitz S, Klinger E, Korczyn AD, Josman N. Use of the virtual action planning supermarket for the diagnosis of mild cognitive impairment. Dementia and geriatric cognitive disorders 2009; 27:301-309. doi: https://doi.org/10.1159/000204915

23. Kizony R, Korman M, Sinoff G, Klinger E, Josman N, Sharky P. (2012). Using a virtual supermarket as a tool for training executive functions in people with mild cognitive impairment, Proc. 9th Intl Conf. Disability, Virtual Reality & Associated Technologies Laval, France, 10–12 Sept. 201.
